# Comparison between stone and digital cast measurements in mixed dentition: Validity, reliability, reproducibility, and objectivity

**DOI:** 10.1007/s00056-022-00376-9

**Published:** 2022-03-03

**Authors:** Lisa Schieffer, Lukas Latzko, Hanno Ulmer, Natalie Schenz-Spisic, Ulrike Lepperdinger, Magdalena Paulus, Adriano G. Crismani

**Affiliations:** 1 University Hospital for Orthodontics, Department of Dental and Oral Medicine and Cranio-Maxillofacial and Oral Surgery, Medical University of Innsbruck, Anichstr. 35, 6020 Innsbruck, Austria; 2 University Hospital for Craniomaxillofacial and Oral Surgery, Department of Dental and Oral Medicine and Cranio-Maxillofacial and Oral Surgery, Medical University of Innsbruck, Anichstr. 35, 6020 Innsbruck, Austria; 3 Department of Medical Statistics, Informatics and Health Economics, Innsbruck Medical University, Schöpfstr. 41/1, 6020 Innsbruck, Austria; 4 University Hospital for Dental Prosthetics and Restorative Dentistry, Department of Dental and Oral Medicine and Cranio-Maxillofacial and Oral Surgery, Medical University of Innsbruck, Anichstr. 35, 6020 Innsbruck, Austria

**Keywords:** Orthodontics, Dental casting technique, Virtual arch models, Three-dimensional imaging, Intraoral scanner, Zahnärztliche Modelltechniken, Virtuelle Bogenmodelle, Dreidimensionale Bildgebung, Intraoraler Scanner

## Abstract

**Purpose:**

To assess the validity, reliability, reproducibility, and objectivity of measurements on stone casts of patients with mixed dentitions compared to measurements on three-dimensional (3D) digital models derived from surface scans of the stone casts.

**Methods:**

Pairs of stone casts of 30 young patients in their mixed dentition stage were included and processed into 3D digital models using an intraoral scanner (iTero Element 2; Align Technology, San Jose, CA, USA). Then an experienced and an inexperienced examiner independently performed measurements of five defined parameters, each in triplicate, both on the digital models with analysis software (OnyxCeph3™; Image Instruments, Chemnitz, Germany) and on the original casts with a vernier calliper. Paired *t*-tests were used for validity and interexaminer objectivity, Pearson correlation coefficients for intermethod reliability, and intraclass correlation coefficients (ICCs) for reproducibility testing.

**Results:**

Significant (*p* < 0.05) intermethod differences were identified for four parameters, but only the differences for overbite and intermolar distance exceeded the threshold of clinical relevance (≥ 0.5 mm). Intermethod reliability was high and method error invariably lower for the digital measurements and for the experienced examiner. Both examiners achieved ICCs > 0.907 with both methods. Interexaminer variation involved significant differences for all parameters but one (intermolar distance) on the stone casts and for three parameters on the digital models.

**Conclusion:**

Measurements performed on digital models of mixed dentitions can yield clinically acceptable outcomes with OnyxCeph3™ software. Both the digital and the analogue measurements were highly reproducible and reliable. Objectivity of the measurements could not be confirmed, as operator experience did make a difference.

## Abstract (de)

**Ziel:**

Ziel der Studie war es, einen Methodenvergleich zwischen manueller und digitaler Vermessung an Modellen des Wechselgebisses vorzunehmen. Bewertet werden sollten die Gütekriterien der Validität, Reliabilität, Wiederholbarkeit und Objektivität.

**Material und Methode:**

In die Studie eingeschlossen wurden 30 Gipsmodelle von Patient:innen mit Wechselgebiss. Die Gipsmodelle wurden mit Hilfe eines Intraoralscanners (iTero Element 2; Align Technologiy, San Jose, CA, USA) digitalisiert. Zwei unabhängige Untersuchende führten die Modellvermessungen (5 Parameter) jeweils 3‑mal an jedem Modell durch. Dabei verwendete man eine Schiebelehre für die Vermessung der Gipsmodelle und eine Computersoftware (OnyxCeph3™; Image Instruments, Chemnitz, Deutschland) für die Vermessung der virtuellen Modelle. Gepaarte t‑Tests wurden für die Bewertung von Validität und Objektivität herangezogen, der Pearson-Korrelationskoeffizient für die Reliabilität und der Intraklassen-Korrelationskoeffizient (ICC) für die Wiederholbarkeit.

**Ergebnisse:**

Signifikante (*p* < 0,05) Methodenunterschiede wurden bei 4 gemessenen Parametern beobachtet, jedoch überstieg lediglich die Differenz für Überbiss und Intermolarenabstand die klinische Relevanz (≥ 0,5 mm). Die Reliabilität beider Methoden war gegeben und der Methodenfehler zeigte sich für die digitale Messmethode und den erfahrenen Befundenden stets niedriger. Beide Befundenden erreichten ICCs > 0,907 für beide Methoden. Signifikante Unterschiede zeigten sich zwischen den beiden Befundenden in allen Parametern außer der Intermolarendistanz bei der Vermessung der Gipsmodelle und in 3 Parametern nach der digitalen Auswertung.

**Conclusio:**

Die digitale Vermessung von Wechselgebissen erzielt klinisch verwertbare Ergebnisse, wenn sie mit Hilfe von OnyxCeph3™ durchgeführt wird. Beide Messmethoden zeigten eine hohe Reliabilität und Wiederholbarkeit. Jedoch spielt die Erfahrung des Befundenden eine wichtige Rolle.

## Introduction

Orthodontics is going digital. In recent years, digitization has become an indispensable part of orthodontists’ workflows. Paperless patient information systems, including digital photographs and radiographs, are today standard [33].

In addition, it is essential for comprehensive orthodontic diagnostics and treatment planning to analyse study casts that represent the patient’s dentition. Mixed dentitions require high accuracy in evaluating arch length discrepancies (ALD) for successful orthodontic treatment, and space analysis in these situations is traditionally performed by contrasting the existing mesiodistal width of the supporting area with a nominal value [27].

The gold standard for diagnostic measurements is to use a calliper on a stone cast [3]. Study casts made of dental stone are, however, susceptible to damage and loss of accuracy by external forces [10]. Following research and development to address this issue, Cadent (now Align Technology, San Jose, CA, USA) introduced the first generation of OrthoCad™ software for “digital casts” in 1999 [15]. In 2006, this was followed by the iTero Element (Align Technology, San Jose, CA, USA) intraoral scanner (IOS), which uses parallel confocal imaging and point-and-stitch reconstruction to generate three-dimensional (3D) computerized images. Current software applications (e.g., OnyxCeph3™; Image Instruments, Chemnitz, Germany) are capable of managing, analysing, and generating virtual arch models.

Reported benefits of using an IOS include better patient comfort [43], reduced storage requirements for casts [29], ability to share data easily anywhere in the world [39], extensive possibilities of analysing digital models [39], and time efficiency [14, 26]. As a result, 3D imaging is today routinely used in orthodontic diagnostics and treatment planning. Young patients, in particular, tend to require maximum comfort and hence prefer the digital impression technique [5]. Digital study models can be generated in three different ways: directly by intraoral scanning, indirectly by surface scanning of stone casts, or based on a cone-beam computed tomography (CBCT) scan [40].

Previous studies have confirmed the utility of intraoral scanning in orthodontic diagnostics and planning [1, 2, 19]. Digital models obtained by extraoral scanning of stone casts have also been shown to offer both high accuracy compared to stone casts per se [10, 31, 42] and adequate precision for orthodontic applications [15, 39]. Numerous studies have confirmed the validity [2, 4, 14, 21, 28, 29, 42, 45], the reliability [4, 14, 15, 20, 21, 24, 34, 42], and the reproducibility [36, 42, 45] of measurements performed on digital models versus on stone casts in permanent dentitions. Despite some findings of statistically significant differences between the methods, these measurement differences were clinically not relevant [7, 8, 20, 23, 24, 30, 34, 36]. Studies have also verified that digital measurements are clinically acceptable and are not inferior for treatment planning [30, 36, 41].

However, while the digital method is a clinically acceptable alternative to the analogue gold standard in analysing permanent dentitions, no comparative studies have been available for digital versus analogue measurements in children with mixed dentitions. These latter situations are different in that, rather than measuring all teeth, a limited number of permanent teeth need to be analysed along with the supporting area. Hence, longer distances must be measured when examining the supporting area, which might be more difficult to achieve accurately.

Evidence-based orthodontics relies on analysis methods known to offer *validity, reliability, reproducibility*, and *objectivity* for treatment planning to remain unaffected. The aim of validation is to prove that these requirements are met by an intended analysis technique in daily practice [39]. In this context,* validity* is defined as the truth value of evidence and whether what is measured equals what was intended to be measured, equating accuracy [35], *intermethod reliability *as the degree to which test scores are consistent when there is variation in the methods or instruments used [13] and *reproducibility*, being concerned with the consistency of evidence [12], as the closeness of agreement between the results of successive measurements of the same measure carried out under the same conditions [44].

Against this background, we designed the present study to assess measurements obtained on digital models as compared to the gold standard. Given the variance in how well a method performs depending on the experience of the examiner, we incorporated this factor into the study design to assess *objectivity*, defined as neutrality of evidence [12].

The null hypothesis of the study was that measurements on digital models would not show significant differences from that on conventional stone casts so that the digital method will be considered as accurate, reliable, reproducible, and objective.

## Methods

Approval of the study design, which was in accordance with the 1975 Declaration of Helsinki as revised in 2013, was obtained by the Ethics Committee of the Medical University Innsbruck (Innsbruck, Austria; study ID: 1124/2021). Using G*power software (version 3.0.1; University of Düsseldorf, Germany), a sample size estimation was conducted as described elsewhere [16, 23, 25] to compare differences between the dependent groups by paired *t*-tests. Assuming mean differences of 0.5 mm with standard deviations < 1 mm as clinically relevant, a sample size of 30 pairs of stone casts was found to be required to achieve a power of 80%, with α set to 0.05 and β to 0.2.

Hence, we randomly retrieved 30 pre-existing pairs (upper and lower arch) of stone casts that represented mixed dentitions from the records of our Department of Orthodontics (Medical University of Innsbruck, Austria). All had been fabricated from ISO type 4 stone (Silky-Rock yellow; Whip Mix, Louisville, KY, USA). Based on dental age, we estimated that these fully anonymized orthodontic patients had been 7–13 years old.

We gathered this sample based on the criteria of mixed dentition (permanent incisors and first molars fully erupted), intact stone casts, and no previous orthodontic treatment. To ensure homogeneity of the sample, care was taken choosing only casts with fully erupted teeth. Exclusion criteria were syndromes.

**Fig. 1:**
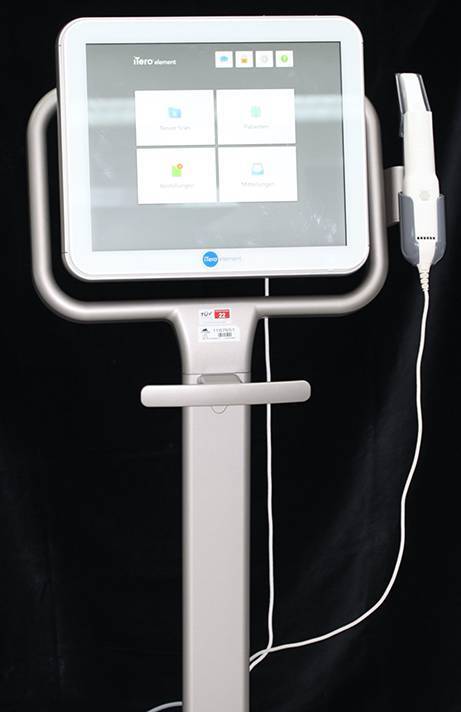
Intraoral scanner (iTero Element 2; Align Technologies) Intraoralscanner (iTero Element 2; Align Technologies)

Baseline measurements on the casts were performed using a vernier calliper (Zurich model; Dentaurum, Ispringen, Germany) to the nearest 0.1 mm. Then, the casts were mounted in a stand and digitized by one experienced operator using an IOS (iTero Element 2, version 1.12.0.990; Align Technology) as recommended by the manufacturer (Fig. 1). All scans were visually checked on screen, followed by re-scanning whenever a flaw was identified. Upper and lower casts were scanned separately, the scans saved as standard tessellation language (STL) files and uploaded to the internet for further transformation into digital casts.

**Fig. 2:**
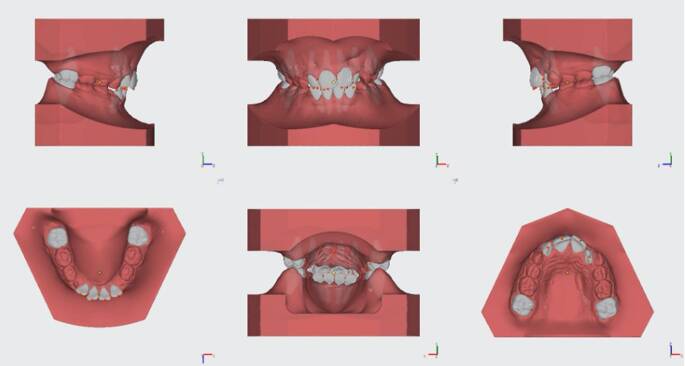
Screenshots of a digital model stored and measured in OnyxCeph3^TM^ (Image Instruments) software Screenshots eines digital vermessenen Modells, Software OnyxCeph3^TM^ (Image Instruments)

**Fig. 3:**
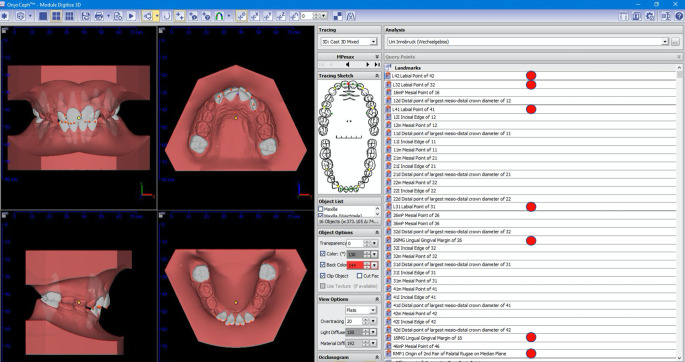
A digital study cast portrayed in OnyxCeph3^TM^ (Image Instruments) dental software with measuring points set. The *red dots* indicate the landmarks which must be set by the operator Ein digitales Modell im OnyxCeph3^TM^ (Image Instruments) Bearbeitungsprogramm. In *rot* jene Punkte, die vom Befundenden selbst gesetzt werden müssen

All of the returned digital casts, accessible for download within 48 h, were measured applying the OnyxCeph3™ Pro analysis software (version 3.2.147; Image Instruments, Chemnitz, Germany) on the same computer (Fig. 2). This environment supports an effective workflow for orthodontic archiving, diagnostics, planning, and counselling, thus assisting orthodontists in optimizing administrative tasks and implementing treatment. For the segmentation process, the middle of each tooth must be selected by the examiner to simulate the shape of each tooth. Then, the software offers automated identification of 20 landmarks (12 mesiodistal widths of incisors and molars, 8 upper and lower incisal edges) and requires input for seven more points, including one point on the palatal aspect of each upper molar, one on the labial aspect of each lower incisor, and the origin of the second pair of palatal rugae on the median plane (Fig. 3).

To assess objectivity, two examiners with different levels of experience performed the same measurements independently: an orthodontist with four years of practice and experienced in scanning (examiner 1), and a dental student in his last year of training without any experience in orthodontics or scanning (examiner 2). To calibrate reproducibility and minimize interexaminer variability, all measurements on stone casts and digital models were performed in triplicate by both examiners under identical conditions with intervals of at least one week.

To evaluate the validity, measurements for maxillary and mandibular space analysis were performed to the nearest 0.1 mm, recording and calculating for each model the amounts of available space, required space, and arch length discrepancy (ALD). Overjet, overbite, and upper intermolar distance were measured to the nearest 0.5 mm: overjet from the incisal edge of the most anterior upper incisor to the labial surface of the most anterior lower incisor; overbite as the longest vertical overlap between upper and lower incisors; maxillary intermolar distance (McNamara, [22]) between the lingual surfaces of both upper first molars. Clinical relevance was assessed by subtracting the mean differences of repeated measurements obtained on the stone casts from those on the digital models. Positive values of mean differences (∆) indicated that the measurements on the stone casts were smaller than those on the digital models. In accordance with the literature, differences ≥ 0.5 mm were regarded as clinically relevant [34].

For validity assessment, only measurements of examiner 1 were taken into consideration, in order to avoid distorted results due to inexperience.

## Statistics

Measurements were entered in a spreadsheet (Microsoft Excel, Redmond, WA, USA) and analysed in SPSS Statistics (v. 26; IBM SPSS Inc., Armonk, NY, USA). Displaying the variables along with their mean values (trueness) and standard deviations (precision), a Shapiro–Wilk test was used to check for normal distribution and Levene’s test for homogeneity of variance. Additionally, 95% confidence intervals (CIs) were calculated. Mean repeated measurements for each examiner and method were compared using paired *t*-tests for validity and objectivity. Method errors were evaluated based on Dahlberg’s formula and intermethod reliability by Pearson correlation coefficients (r). Reproducibility was assessed as intra-examiner repeatability of measurements, using intraclass correlation coefficients (ICCs), > 0.9 indicating excellent, 0.75 to < 0.9 good, 0.5 to < 0.75, moderate, and < 0.5 poor reproducibility. Differences emerging from any of these statistical tests were regarded as significant at *p* < 0.05 or as highly significant at *p* < 0.01.

## Results

### Validity

Table 1 summarizes the mean values, standard deviations, and the corresponding 95% confidence intervals (CI) for all measurements performed by the experienced examiner. Comparing the mean values obtained for each of the five parameters measured (each mean value representing three repeated measurements performed on either the stone casts or the digital models), we observed differences of +0.49 mm for ALD upper jaw (UJ), −0.44 mm for ALD lower jaw (LJ), +1.04 mm for overbite, −0.45 mm for overjet, and +1.18 mm for McNamara. With the exception of ALD UJ, all these differences between both methods were significant (*p* < 0.05) or highly significant (*p* < 0.01). However, only the parameters of overbite and McNamara exceeded the threshold of clinical significance (≥ 0.5 mm) with differences of 1.04 and 1.18 mm, respectively (Table 1). The differences for ALD UJ, ALD LJ, and overjet were not clinically relevant (< 0.5 mm).

**Table 1:** Validity expressed as between-method differences in the hands of the experienced examiner Validität ausgedrückt als Unterschiede zwischen den Methoden, durchgeführt vom erfahrenen Untersuchenden

	Stone casts (*n* = 30)			Digital models (*n* = 30)				Paired *t*-test (*p*)
Parameter	Mean	SD	CI	Mean	SD	CI	∆	
ALD UJ (mm)	0.82	2.13	0.02–1.62	0.33	2.85	−0.73–1.39	+0.49	0.28
ALD LJ (mm)	0.47	3.17	−0.71–1.65	0.91	2.98	−0.20–2.02	−0.44	< 0.05*
Overbite (mm)	3.43	1.81	2.75–4.11	2.39	1.82	1.71–3.07	+1.04	< 0.01**
Overjet (mm)	4.69	2.49	3.76–5.62	5.24	2.33	4.37–6.11	−0.45	< 0.01**
McNamara (mm)	33.52	1.75	32.87–34.17	32.34	2.05	31.57–33.11	+1.18	< 0.01**

*ALD* arch length discrepancy, *UJ* upper jaw, *LJ* lower jaw, *SD* standard deviation, *CI* confidence intervals, *∆* difference of means from deviations

*Difference statistically significant at *p* < 0.05, **difference highly significant at *p* < 0.01

Thus, the null hypothesis regarding the accuracy of measurements obtained from digital casts had to be rejected, at least partly.

### Intermethod reliability

Table 2 lists both examiners’ random errors of repeated measurements. On the stone casts, the experienced examiner 1 produced errors within a range of 0.03 to 0.53 mm, while the inexperienced examiner 2 had a considerably harder time, with errors ranging from 0.1 to 0.75 mm. While both did invariably better on the digital models, examiner 2 again incurred higher errors for all five parameters. Given r‑values of 0.499–0.918, a positive correlation between both measuring methods could be established for all parameters (Table 2).

**Table 2:** Intermethod reliability expressed as measurement errors calculated by Dahlberg’s formula for each examiner and as between-method correlations Inter-Methoden-Reliabilität ausgedrückt als Messfehler, berechnet nach der Dahlberg-Formel für jeden Untersuchenden und als Korrelationen zwischen den Methoden

	Stone casts (*n* = 30)		Digital models (*n* = 30)		PCC (r)
Parameter	Examiner 1 Dahlberg	Examiner 2 Dahlberg	Examiner 1 Dahlberg	Examiner 2 Dahlberg	
ALD UJ (mm)	0.53	0.56	0.07	0.25	0.499**
ALD LJ (mm)	0.25	0.74	0.13	0.19	0.918**
Overbite (mm)	0.03	0.17	0.02	0.06	0.836**
Overjet (mm)	0.06	0.1	0.04	0.07	0.826**
McNamara (mm)	0.18	0.23	0.08	0.14	0.806**

*ALD* arch length discrepancy, *UJ* upper jaw, *LJ* lower jaw, *PCC* Pearson correlation coefficient

**Difference highly significant at *p* < 0.01

Based on these results, the null hypothesis had to be accepted.

### Reproducibility

Table 3 gives an overview of intraclass correlation coefficients, which were calculated as a measure of consistency which the experienced and the inexperienced examiner achieved in reproducing their results over repeated measurements. By and large, both examiners achieved high ICCs on the stone casts and on the digital models. The lowest ICCs associated with any of the parameters measured, regardless of the measuring methods, were 0.918 for examiner 1 and 0.666 for examiner 2. Overall, therefore, 0.666 was the lowest ICC. Examiner 1 was most consistent with the transversal measurement according to McNamara on the stone casts (ICC: 0.998) and with overbite on the digital models (ICC: 0.999) versus least consistent with ALD UJ (ICC: 0.918) and McNamara (ICC: 0.968), respectively. Examiner 2 was most consistent with overjet on the stone casts (ICC: 0.975) and ALD LJ on the digital models (ICC: 0.953) versus least consistent with overbite (ICC 0.931) and McNamara (ICC 0.907), respectively (Table 3).

**Table 3:** Reproducibility expressed as within-examiner agreement of repeated measurements Reproduzierbarkeit ausgedrückt als prüferinterne Übereinstimmung wiederholter Messungen

	Stone casts (*n* = 30)	Digital models (*n* = 30)	Stone casts (*n* = 30)	Digital models (*n* = 30)
Parameter	Examiner 1, ICC		Examiner 2, ICC	
ALD UJ	0.918	0.986	0.666	0.875
ALD LJ	0.952	0.987	0.934	0.953
Overbite	0.999	0.969	0.931	0.929
Overjet	0.982	0.979	0.975	0.944
McNamara	0.998	0.968	0.952	0.907

*ALD* arch length discrepancy, *UJ* upper jaw, *LJ* lower jaw, *ICC* intraclass correlation coefficient

Overall, the null hypothesis regarding the reproducibility of measurements for both methods had to be verified.

### Objectivity

Table 4 summarizes all mean values of measurements and the calculated interexaminer differences, evaluated both by paired *t*-tests to determine statistical significance and by absolute differences to determine clinical relevance. The method on stone casts was associated with significant (*p* < 0.05) or highly significant (*p* < 0.01) differences for all parameters except the transversal measurement according to McNamara (*p* = 0.12). None of these differences exceeded the 0.5 mm threshold of clinical relevance. The interexaminer differences on digital models were significant for three parameters (ALD LJ, overbite, McNamara; *p* < 0.01), but only one (McNamara: −1.83 mm) was clinically relevant (Table 4).

**Table 4:** Objectivity expressed as between-examiner differences identified for each method Objektivität ausgedrückt als Unterschiede zwischen den Prüfenden, die für jede Methode identifiziert wurden

	Stone casts (*n* = 30)			Paired *t*-test	Digital models (*n* = 30)			Paired *t*-test
Parameter	Examiner 1 Mean	Examiner 2 Mean	∆	*p*-value	Examiner 1 Mean	Examiner 2 Mean	∆	*p*-value
ALD UJ (mm)	0.82	0.68	+0.14	< 0.01**	0.33	0.12	+0.21	0.08
ALD LJ (mm)	0.47	0.03	+0.44	< 0.01**	0.91	0.48	+0.43	< 0.01**
Overbite (mm)	3.43	3.34	+0.09	< 0.05*	2.39	2.46	−0.07	< 0.01**
Overjet (mm)	4.69	4.43	+0.26	< 0.01**	5.24	5.31	−0.07	0.29
McNamara (mm)	33.52	33.59	−0.07	0.12	32.34	34.17	−1.83	< 0.01**

*ALD* arch length discrepancy, *UJ* upper jaw, *LJ* lower jaw, *∆* difference of means from deviations

*Difference statistically significant at *p* < 0.05, **difference highly significant at *p* < 0.01

In consideration of these findings, the null hypothesis concerning the objectivity, had to be declined.

## Discussion

The authors are not aware of any previous studies evaluating the validity, reliability, reproducibility, and objectivity of measurements performed on digital models of mixed dentitions. Because there is no direct way of determining the true dimensions of teeth, measurements on stone casts were considered the gold standard against which validity was to be evaluated.

Since the accuracy of digital models is known to depend on scanner types and intraoral conditions (e.g., saliva or restricted mouth opening) [6, 11], our use of surface scans performed on stone casts ruled out any loss of precision that may have been incurred by clinical intraoral capturing. In other words, none of the differences between stone casts and digital models we observed can be attributed to intraoral scanning distortion.

In the hands of the experience examiner, all mean differences in repeated measurements between the stone casts and the digital models were significant (*p* < 0.05) or highly significant (*p* < 0.01), the only exception being ALD UJ (*p* = 0.28). However, clinically relevant differences were noted for only two parameters (overbite: 1.04 mm; transversal measurement according to McNamara: 1.18 mm). Differences in overbite measurements were most likely due to the delicate contact points on the digital images compared to the bulky calliper on the stone casts. Differences noted between the analogue and digital measurements of intermolar distance might be a function of the longer distance to be measured.

For three out of five measured parameters, our results, although based on a different method of digitization and different software (OnyxCeph3™), support previous findings that the validity of measurements on digital models (OrthoCad) is clinically acceptable [4, 20, 21, 28]. Radeke et al. [29], who previously compared measurements on stone casts and digital models with OnyxCeph3™, did report differences between software- and calliper-based measurements of mesiodistal tooth width but found them to be operator-associated and not statistically significant. Zilberman et al. [45] and Santoro et al. [34] found that stone casts resulted in slightly better measurements than digital models. Both studies relied on impressions sent to OrthoCad, so that any flaws during impression-taking or subsequent alginate shrinkage could have been passed on to the digital models. Alginate shrinkage has been previously reported to account for underestimated measurements on digital models, compared to the situation in vivo [24, 37], which might affect the validity.

Consistent with our own result that manual overbite measurements were significantly increased (Table 1), the manual method has repeatedly been implicated in exaggerated overbite measurements [34, 38]. Thus, in absolute terms of millimetres, smaller teeth should be expected to result in smaller overbite values, although inconsistent overbite measurements could also have been introduced by other factors such as incorrect probe angulation during manipulation of the traditional casts or rounding of the digital measurements to the nearest 0.5 mm. Another explanation might lie in digital measurements of tooth size consistently falling short of the values obtained on stone casts [23, 36]. In our study, this observation held true for the parameters ALD UJ, overbite, and the transversal measurement according to McNamara.

Notwithstanding, it stands to reason that the measuring tools of the OnyxCeph3™ software may allow for better defined measuring protocols resulting in more valid measurements than callipers used on stone casts. Further, automated identification of many landmarks requires the examiner merely to visually check these results (Fig. 3). Thus, one could speculate that inaccuracies of analogue measurements may be introduced by human error in the form of misreading callipers or misplacing reference points. That said, only the reference points for ALD UJ, ALD LJ, and overbite measurements are automatically set by OnxyCeph3^TM^, whereas analogue versus digital analysis yielded significantly different mean values for all the parameters investigated but one (ALD UJ: *p* = 0.28).

Intermethod reliability indicates the agreement of results obtained with any two comparable measuring methods. A Pearson correlation coefficient of > 0.499 testifies to a high degree of agreement between the two methods in our study (Table 2). Zilberman et al. [45] and Bell et al. [2] reported mean intra-examiner errors of 0.18 or 0.17 mm, indicating excellent results on both stone casts and digital models in line with our own findings.

Intra-examiner ICC values as calculated in the present study yield estimates of reproducibility under the model of equal marginal distributions. When marginal distributions are inaccurate, the deviations are captured and rated as unreliable. Based on all parameters, consistent agreement of repeated measurements on the stone casts emerged for the experienced examiner 1, given an ICC > 0.918 as compared to > 0.666 for the inexperienced examiner 2 (Table 3). All parameters measured by examiner 1 achieved ICCs > 0.9 (= excellent reproducibility). Most of those performed by examiner 2 also exceeded the threshold of excellent reproducibility; one digitally measured parameter was rated as “good” (ICC: 0.8775) and one analogue parameter as “moderate” (ICC: 0.666). Overall, both examiners achieved very good ICCs. The fact that those for the experienced examiner were slightly higher suggest that different levels of orthodontic experience may have an influence on reproducibility.

The reproducibility of analogue measurements performed on stone casts may not only depend on an operator’s ability to identify landmarks and to accurately transfer quantitative data to a computer, but there is a need for careful handling to avoid breakage of the casts. The reproducibility of digital measurements will also depend on the hardware and software used for digitization [18]. In the present study, excellent reproducibility was noted for measurements performed on both stone casts and digital models. Similar results have been reported previously by Wiranto et al. [42], Stevens et al. [36], and Czarnota et al. [8]. Measurements of arch length discrepancy were associated with slightly higher ICCs for the digital technique. Measurements of overbite, overjet, and the transversal measurement according to McNamara involved moderately higher ICCs for the calliper-based analogue technique. Arguably, this might be because the reference points for overjet and McNamara need to be manually selected on screen, thus being more prone to misjudgement, while all landmarks for tooth-width and space analysis are identified by OnyxCeph3™ on its own.

Four out of five interexaminer differences for measurements on stone casts were statistically significant (Table 4). All these differences, however, remained below the threshold of clinical relevance (< 0.5 mm). Based on digital measurements, significant interexaminer disagreement was seen for three parameters (ALD LJ, overbite, transversal measurement according to McNamara) and reached clinical relevance for McNamara (−1.83 mm). Given this nearly complete absence of clinically relevant differences between both examiners, both methods may be regarded as objective. The only exception just mentioned, a large interexaminer difference between the digital measurements of upper intermolar distance, may be attributed to operator experience, as the landmarks for this parameter need to be identified manually in OnyxCeph3™. This finding is consistent with Radeke et al. [29]. Intermolar distance remains a major problem of intraoral scanners, with large deviations incurred by the matching/stitching algorithm [17, 32].

Our data further support findings of Dalstra et al. [9] about the effect that interexaminer variation was generally low. Rheude et al. [30] investigated how orthodontic experience might influence treatment planning. In accordance with our own results, they found statistically significantly differences in diagnostic decisions, which did not, however, result in different treatment decisions. It should be noted that the present study featured an inexperienced examiner solely to identify different measurements as a function of orthodontic expertise.

Limitations to our study arise from the fact that the intraoral scanner was used on stone casts rather than in patients’ mouths and examination was done retrospectively. Though, the sample size was checked conscientiously, it might be limited for a study of diagnostic accuracy.

## Conclusions

Digital models of permanent dentitions are today an accepted alternative to stone casts in orthodontics. Driven by both an emerging aesthetic idealism and intraoral scanners eliminating the need for impression-taking, the demand for orthodontic treatment of paediatric patients has been growing in recent years. Intraoral scanners can minimize discomfort, which is essential in children. This preliminary study confirms that digital models (here generated based on surface scans of physical casts) can be a reliable alternative to stone casts in analysing mixed dentitions. Measurements performed on these virtual models yield reproducible intra- and interarch relationships, but the results are affected by operator experience. We cannot confirm that longer distances have a greater deviation between measurements, even though the transversal measurement according to McNamara is one long distance to measure, but the space analysis requires many small measurements. Well-designed investigations dealing with intraoral scanning followed by digital model generation are needed to assess the time requirements for these procedures in young patients to evaluate the feasibility of this digital work-flow.
